# Dehydroepiandrosterone-induced polycystic ovary syndrome mouse model requires continous treatments to maintain reproductive phenotypes

**DOI:** 10.1186/s13048-023-01299-8

**Published:** 2023-10-25

**Authors:** Haowen Wu, Bining Zhao, Qiyang Yao, Jihong Kang

**Affiliations:** Department of Physiology and Pathophysiology, School of Basic Medical Sciences, Peking University Health Science Center, No.38 Xueyuan Rd, Haidian District, Beijing, 100191 China

**Keywords:** Polycystic ovary syndrome, Phenotype maintenance, Treatment withdrawal

## Abstract

**Background:**

Polycystic ovary syndrome (PCOS) is the most common endocrinopathy associated with infertility and metabolic disorder in women of reproductive age. Animal models have been developed and used as tools to unravel the pathogenesis of PCOS, among which most postnatal models employ continuing experimental manipulations. However, the persistence and stability of these animals after modeling is unknown. Dehydroepiandrosterone (DHEA)-induced PCOS mouse model is commonly used in PCOS studies. Thus the aim of the present study was to investigate the reproductive features of DHEA-induced PCOS mice fed a normal chow or an high-fat diet (HFD) with treatment withdrawal or consecutive treatments after PCOS mouse models were established.

**Methods:**

Prepubertal C57BL/6 J mice (age 25 days) were injected (s.c.) daily with DHEA on a normal chow or a 60% HFD for 20 consecutive days to induce PCOS mouse models. Mice injected with the vehicle sesame oil were used as controls. After 20 days, mice were divided into 2 groups, namely “Continue dosing group” and “Stop dosing group”. The animals were consecutively treated with DHEA or DHEA + HFD, or housed without any treatment for 2 or 4 weeks. Estrous cycles were evaluated during this period. At the end of the experiment, serum testosterone (T) levels were measured and the morphology of ovaries was evaluated.

**Results:**

The mice in Continue dosing groups maintained reproductive phenotypes of PCOS mouse models. In contrast, 2 or 4 weeks after PCOS models were established, the mice with treatment withdrawal in Stop dosing groups exhibited normal serum testosterone levels, regular estrous cycle, and relatively normal ovarian morphology. In addition, even with consecutive treatments, there was no marked difference in body weight between DHEA mice on the normal chow or an HFD in Continue dosing groups and the control animals 3 weeks after modeling.

**Conclusions:**

After PCOS mice were induced with DHEA or DHEA + HFD, the mice still need consecutive treatments to maintain reproductive phenotypes to be regarded as PCOS mice that meet the diagnostic criteria of PCOS defined by the 2003 Rotterdam criteria.

**Supplementary Information:**

The online version contains supplementary material available at 10.1186/s13048-023-01299-8.

## Introduction

Polycystic ovary syndrome (PCOS), one of the most common female endocrine disorders, affects up to 6–10% of women of reproductive age worldwide [1]. According to the 2003 Rotterdam criteria, PCOS is defined when at least 2 of the following 3 features are present, given the exclusion of other etiologies: clinical and/ or biochemical signs of hyperandrogenism, oligo- and/or anovulation, and polycystic ovaries [2]. Meanwhile, PCOS also involves metabolic abnormalities in most cases, including insulin resistance (IR), obesity, type 2 diabetes, dyslipidemia, and increased risk of cardiovascular diseases [3–7].

Although PCOS has been intensively researched for several decades, the etiology of PCOS still remains unknown. To better understand the pathogenesis of this syndrome, animal models have been developed and are widely used in the studies of PCOS. PCOS animal models include genetically manipulated mouse models, prenatal, neonatal, peripubertal, and adult induced PCOS models [8]. A series of methods is used to induce PCOS models, including treatments of animals with androgens, such as testosterone propionate (TP), dihydrotestosterone (DHT), dehydroepiandrosterone (DHEA), or other reagents, including estrogens, anti-Müllerian hormone (AMH), letrozole, etc. [9–11]. Presently, the majority of PCOS animal models are generated by inducing hyperandrogenism, which has been shown to produce models that display reproductive, or reproductive accompanied with metabolic PCOS-like features. Additionally, most of these postnatal manipulation-induced animal models employ discrete or continuing experimental operations.

Among these models, the DHEA-induced PCOS mouse model has been commonly used in the research of PCOS. DHEA, one of the metabolic intermediates in the biosynthesis of androgen, is a very important prohormone that plays a key role through the conversion to testosterone (T) and DHT [12]. Prepubertal female mice are usually exposed to DHEA for 15–35 days to induce PCOS mouse models. Particularly, 20 day-treatment is the mostly used DHEA treatment period in mice [13–19]. DHEA-induced mice exhibit human PCOS characteristics, including hyperandrogenism, acyclicity anovulation, polycystic ovaries, and certain metabolic disorders [9, 20, 21]. In addition, our previous study found that mice treated with the combination of DHEA and a 60% high-fat diet (HFD) exhibited both reproductive disorders and exacerbated metabolic derangements [22]. Whether these mice could maintain the phenotypes after modeling, however, remains unknown.

To evaluate the stability and persistence of DHEA- and DHEA + HFD-induced PCOS mice, we divided the mice into two groups, one with continuing manipulations and the other with treatment withdrawal after PCOS models were established. The reproductive phenotypes of the mice were assessed 2 or 4 weeks after grouping, including estrous cycle, blood testosterone levels, and ovarian morphology. Our data suggested that DHEA- or DHEA + HFD-induced PCOS mice still need continuing treatments after modeling in order to be defined as PCOS animal models, which may provide important matters that should be paid attention to when performing long-term experiments using these models.

## Materials and methods

### Animals and experimental protocols

Female C57BL/6 J mice at the age of 21 days were purchased from *Beijing Hfk Bioscience Co., Ltd* (Beijing, China). All animals were housed at 22°C ± 2°C and acclimated to standard laboratory conditions (12 h light:12 h dark) with free access to rodent food and water. The mice of comparable body weights were divided into 3 groups on postnatal Day 25. Group 1: control group. The mice were fed a normal chow and injected (s.c.) daily with the vehicle sesame oil (0.1 ml per 100 g body weight). Group 2: DHEA group. Mice were fed a normal chow (Medicience Ltd., Jiangsu, China) and injected (s.c.) daily with DHEA (Sigma, SIAL, USA) (6 mg per 100 g body weight) dissolved in 0.1 ml sesame oil (ThermoFisher, MA, USA) as described previously [14, 18, 20, 22]. Group 3: DHEA + HFD. The mice were fed a 60% high-fat diet (HFD) purchased from Medicience Ltd. (Jiangsu, China) and injected (s.c.) daily with DHEA. The nutrition compositions of the two diets are shown in Table S1. All the three groups of mice were treated for 20 consecutive days.

**Fig. 1:**
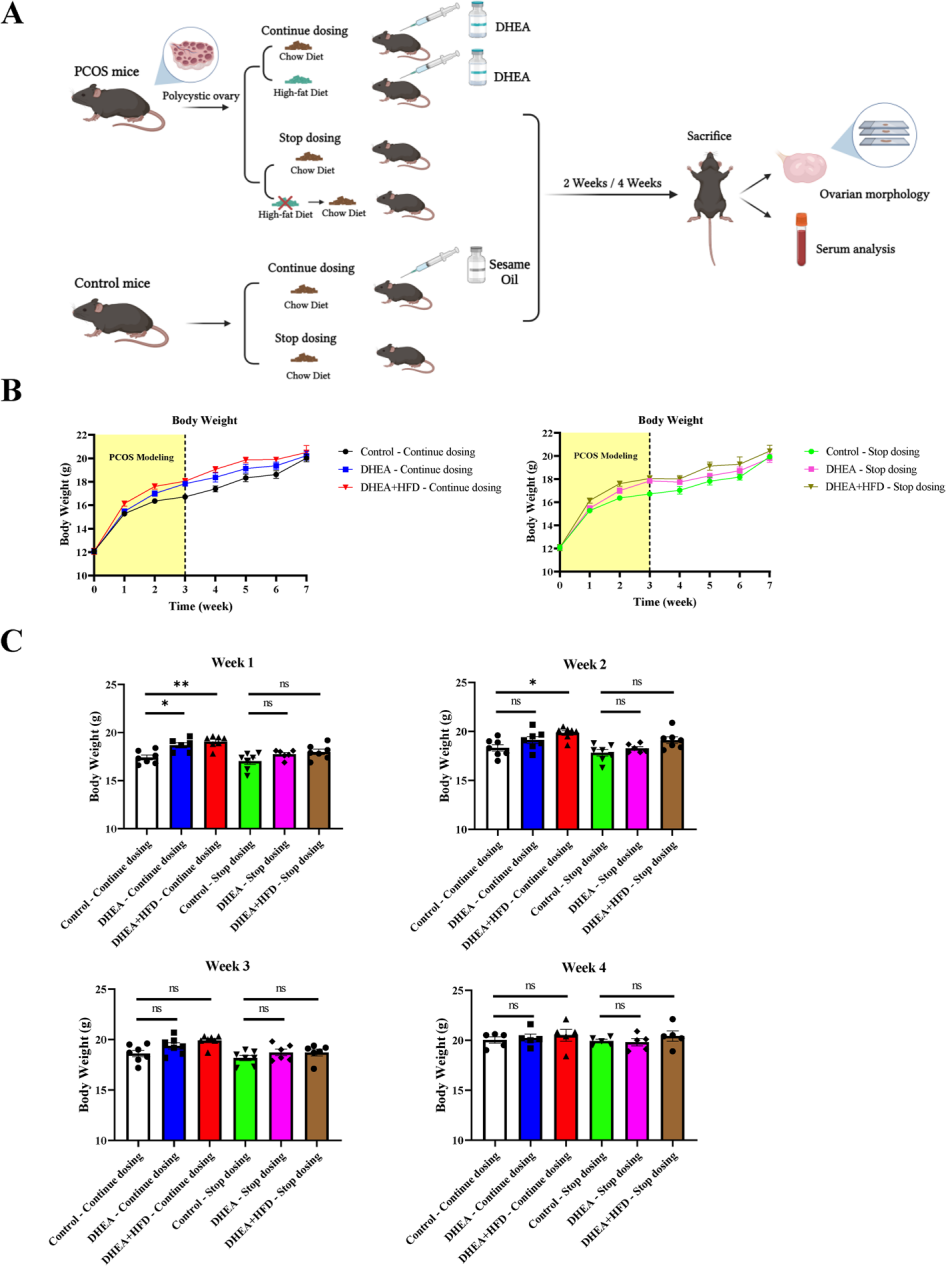
Body weight of the mice from inducing PCOS mouse models to 4 weeks after PCOS mice were induced. **A** Schematic experimental design of DHEA- and DHEA + HFD-induced PCOS mouse models from modeling to 4 weeks after PCOS models were established. **B** Body weight curves of the mice from the beginning of the experiment to 4 weeks after PCOS models were established. Yellow part: the period during PCOS modeling. **C** Body Weight of the mice in each group during different periods after PCOS models were established. Data are presented as mean ± SEM. *P < 0.05, **P < 0.01, *n* = 5–7 per group

PCOS mice were induced after the aforementioned treatments for 20 days, as we reported before [22]. Then each group of mice (Day 45 of life) was divided into 2 groups, namely “Stop dosing group” and “Continue dosing group”. For the mice in Stop dosing groups, all the treatments ended, including sesame oil, DHEA, and HFD. All the mice were housed on a normal chow. For the mice in Continuous dosing groups, the animals were given the same consecutive treatments, including injection (s.c.) daily with sesame oil or DHEA on a normal chow or an HFD, as illustrated in Fig. 1A. 5–7 mice per group were sacrificed 2 or 4 weeks afterward. The body weights of the mice were monitored daily. Reproductive features were evaluated at the end of the experiments. All animal protocols were approved by the Animal Committee of Peking University Health Science Center in accordance with the Guide for the Care and Use of Laboratory Animals published by the US National Institutes of Health.

### Estrous cycle determination

The estrous cycle was determined daily 1 week before the end of PCOS modeling until 2 weeks after modeling, or the last week of the 4-week post-modeling groups. Vaginal cells were collected via saline lavage, dried on a glass slide, and then stained with 0.1% methylene blue staining (Sigma, SIAL, USA). The stages of the estrous cycle were determined based on vaginal cytology: predominant nucleated epithelial cells indicated the proestrus stage, predominant cornified squamous epithelial cells indicated the estrus stage, both cornified squamous epithelial cells and leukocytes indicated the metestrus stage, and predominant leukocytes indicated the diestrus stage.

### Tissue collection and histology

After 2 weeks or 4 weeks post-modeling, the mice were housed for 1 day. Then the mice were deeply anesthetized via an intraperitoneal injection of a mixture of ketamine hydrochloride and xylazine. Blood samples were collected. Serum samples were then separated and kept at -80℃ for further assay. Ovaries were dissected, fixed overnight in 4% paraformaldehyde (PFA) at 4℃, and embedded in paraffin. The fixed ovaries were cut into serial Sects. (6-μm thickness). Every 10th section was mounted on a glass slide and 12 sections from each ovary were used for counting corpus luteum (CL) and cysts. Sections were stained with hematoxylin and eosin (H&E) (Biosharp, Beijing, China) according to the standard histological procedures. Ovarian corpus luteum and cysts were distinguished by histomorphology. Numbers of corpora lutea and cysts were counted.

### Serum testosterone measurement

Serum testosterone levels were measured by ^125^I-labeled radioimmunoassay kits (Beijing North Institute of Biological Technology, China). The within-assay and between-assay variabilities were 10% and 15%, respectively.

### Statistical analysis

Data are presented as mean ± SEM. Statistical analyses were performed with GraphPad Prism 8.0 software. Effects of the treatments were analyzed by one-way analysis of variance (ANOVA) followed by the Bonferroni posttest. *P* < 0.05 was considered statistically significant.

## Results

### Body weight of the mice

The body weight of all the mice was monitored every week for 5 or 7 weeks in total. The results of weekly body weights of the mice in Continue dosing groups and Stop dosing groups were shown in Fig. 1B and C, respectively. The curves in the yellow areas in Fig. 1B indicate the body weight of the control, DHEA, and DHEA + HFD mice during PCOS modeling (from Weeks 1 to 3 of the treatments), whereas curves in the blank areas present the body weight of three groups of mice after PCOS modeling (from Weeks 4 to 7 of the treatments). At the end of PCOS modeling (Week 3), the body weight of the DHEA and DHEA + HFD mice was significantly higher than that of the control animals.

After PCOS mice were induced, each group of mice was divided into Continue dosing group and Stop dosing group. As illustrated in Fig. 1C, the body weight of the DHEA and DHEA + HFD mice in Continue dosing groups were significantly higher than that of the control animals (*P* < 0.05 and *P* < 0.01, respectively) one week after PCOS modeling. Two weeks after PCOS modeling, there was a significant difference in body weight between DHEA + HFD mice and control mice in Continue dosing groups. No marked difference, however, was observed between DHEA mice and control mice in Continue dosing groups since Week 2 to Week 4. For mice in Stop dosing groups, all the treatments were withdrawn after PCOS mice were induced and all the animals were just housed on a normal chow. From Week 1 to Week 4 after PCOS modeling, there was no significant difference in body weight among control, DHEA, and DHEA + HFD mice in Stop dosing groups (Fig. 1C). These results suggested that the difference in body weight among control mice, DHEA- or DHEA + HFD-induced PCOS mice was due to the differential treatments during modeling. In addition, data from Continue dosing groups indicated that even with consecutive treatments with DHEA or DHEA + HFD after PCOS modeling, the body weight of DHEA or DHEA + HFD mice was no longer higher than control mice 2 weeks after PCOS modeling.

### Estrous cycle of the mice

**Fig. 2:**
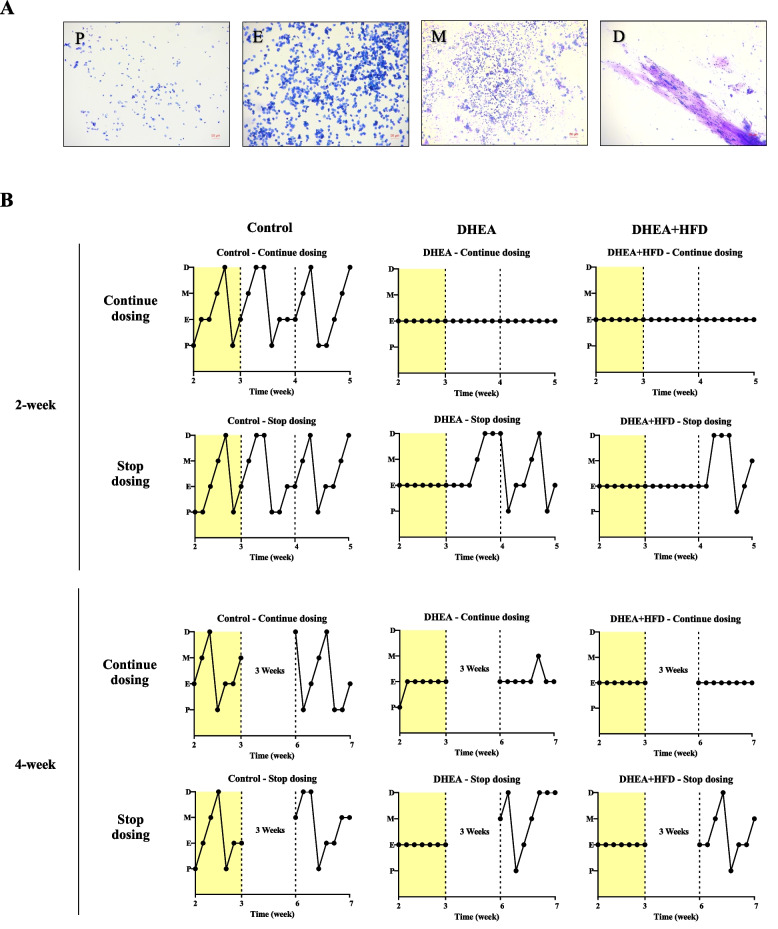
Estrous cycle of the mice. **A** Representative photomicrographs of the four estrus stages of the estrous cycle of a female mouse. Bars = 50 μm. **B** Representative estrous cycle of one mouse from each group. The dashed lines separated the estrous cycle 1 week before the end of modeling (yellow part) from 2 weeks after models were established, or the last week of the 4-week experimental groups. P, proestrus; E, estrus; M, metestrus; D, diestrus

Menstrual disturbance is a typical feature of women with PCOS in clinic. The estrous cycle of mice is defined into 4 stages: proestrus stage, estrus stage, metestrus stage, and diestrus stage (Fig. 2A). Five mice per group were conducted for estrous cycle assay. The estrous cycles of mice were monitored from 1 week before the end of modeling to 2 weeks after PCOS modeling in the 2-week Continue dosing groups and Stop dosing groups. For the mice in the 4-week Continue dosing groups and Stop dosing groups, the estrous cycles of mice were monitored during the last week of the experiment. As illustrated in Fig. 2B, the control mice had normal estrous cyclicity during modeling (yellow areas), 2 weeks, and 4 weeks after modeling. In contrast, DHEA- and DHEA + HFD-induced PCOS mice showed a disturbed estrous cycle during modeling (yellow areas). When PCOS mice in Continue dosing groups received continuous treatments with DHEA or DHEA + HFD after modeling for 2 or 4 weeks, all the mice maintained disrupted estrous cycle (Fig. 2B). However, when the treatments were withdrawn for 1 week after modeling, all the DHEA mice and 60% of DHEA + HFD mice in Stop dosing group exhibited normal estrous cycle, as shown in Table 1. And all the DHEA + HFD mice in Stop dosing groups showed normal estrous cyclicity 2 weeks after modeling (Table 1). These results suggested that DHEA- or DHEA + HFD-induced PCOS mouse models need continuous treatments after modeling to maintain the disturbed estrous cycle.

**Table 1:** The ratio of mice with the normal estrous cycle in each group (%)

During Time^a^	Experimental Groups^b^					
	Control Continue dosing	DHEA Continue dosing	DHEA + HFD Continue dosing	Control Stop dosing	DHEA Stop dosing	DHEA + HFD Stop dosing
Week 1	100	0	0	100	100	60
Week 2	100	0	0	100	100	100
Week 4	100	0	0	100	100	100

^a^Maintenance periods after PCOS modeling

^b^*n* = 5 in each group

### Ovarian function of the mice

**Fig. 3:**
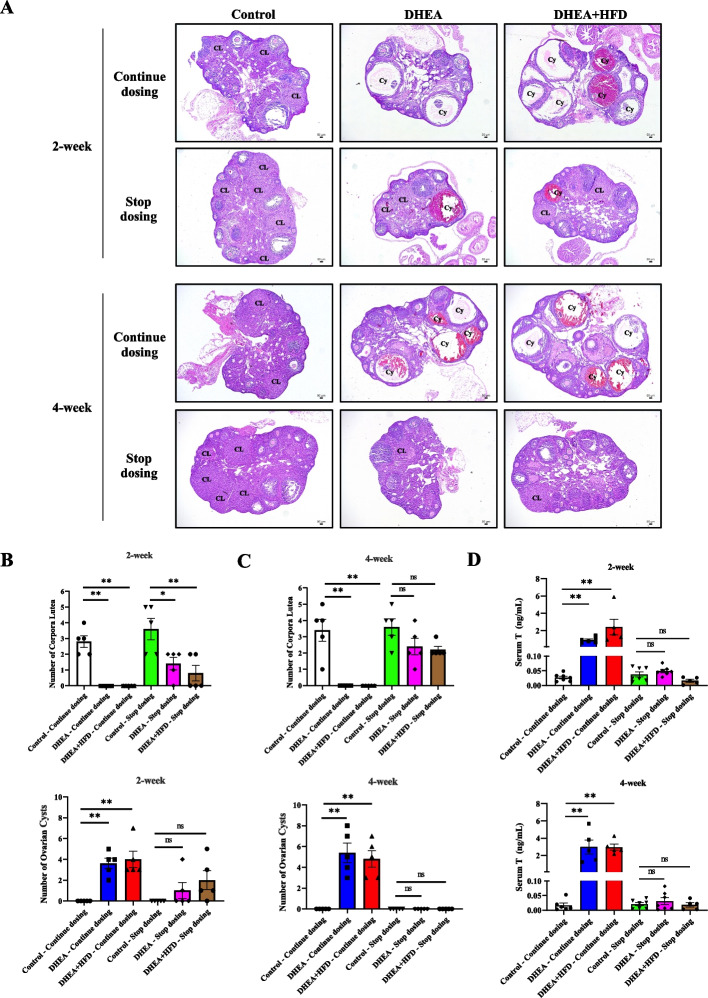
Ovarian morphology and serum testosterone (T) levels of the mice. **A** Representative H&E staining of ovarian sections from one mouse in each group. Bars = 50 μm. CL, corpus luteum; Cy, ovarian cysts. **B** The numbers of ovarian corpus luteum and ovarian cysts in the mice of 2-week experimental groups after models were established. **C** The numbers of ovarian corpus luteum and ovarian cysts in the mice of 4-week experimental groups after models were established. **D** Serum T levels in the mice of the 2-week and 4-week experimental groups after models were established. Data are presented as mean ± SEM. *P < 0.05, **P < 0.01. **B**-**D**, *n* = 5 per group

In DHEA- or DHEA + HFD-induced PCOS mice, visible cysts often appear in the ovaries, accompanied by a decrease in the number of corpus luteum, indicating abnormal ovulation in these mice. Representative micrographs of ovarian sections are shown in Fig. 3A. For all the DHEA and DHEA + HFD mice in Continuous dosing groups at 2 or 4 weeks after modeling, the ovaries of the mice still present typical PCOS ovarian morphology, with remarkable ovarian cysts and lack of corpus luteum (Fig. 3B and C). In contrast, there was no difference in the number of cysts between DHEA or DHEA + HFD mice with control mice in Stop dosing groups 2 weeks after PCOS modeling even though the number of corpus luteum was significantly lower than controls (Fig. 3B). For the DHEA and DHEA + HFD mice in Stop dosing groups 4 weeks after PCOS modeling, there was no difference in the number of corpus luteum or cysts compared with control mice (Fig. 3C). These data indicated that without consecutive treatments after PCOS modeling, DHEA- or DHEA + HFD-induced PCOS mice did not maintain ovarian abnormality.

### Serum testosterone (T) level of the mice

According to Rotterdam criteria, high serum T level is an important diagnostic criterion of the human PCOS. Therefore, serum T levels of all the mice were measured when the animals were sacrificed 2 or 4 weeks after PCOS modeling. For the mice in Continuous dosing groups, serum T levels of the DHEA and DHEA + HFD mice were significantly higher than controls (Fig. 3D). In contrast, there was no marked difference between DHEA or DHEA + HFD mice and control mice in Stop dosing groups 2 weeks or 4 weeks after PCOS modeling (Fig. 3D), suggesting that DHEA- or DHEA + HFD-induced PCOS mice need consecutive treatments after modeling to keep significantly elevated serum levels of T.

## Discussion

Since PCOS has been first described in 1935, numerous animal models were used to mimic this human syndrome. Among these animal models, rodents are the most widely used. Most postnatal manipulation-induced rodent models need discrete or continuing experimental operations. After PCOS models are induced, however, whether or how long these animals can maintain PCOS phenotypes remain unknown. It is thus important to unravel this issue, especially for long-term trials that aim to verify the therapeutic effects of treatments using these continuing experimental operation-induced PCOS animal models. DHEA-induced PCOS mice on a normal chow or an HFD are widely used in PCOS studies. These mice exhibit irregular estrous cycle, high serum levels of T (hyperandrogenism), and ovarian cysts, and are thus recognized as PCOS mouse models according to the 2003 Rotterdam criteria [2]. The difference between DHEA- and DHEA + HFD-induced mice lies in the exacerbated metabolic disorders in DHEA + HFD-treated mice compared with DHEA mice. In the present study, we thus use DHEA- and DHEA + HFD-induced PCOS mice as examples of continuing experimental operation-induced PCOS rodent models. After PCOS mice were induced with DHEA or DHEA + HFD treatments, we divided these mice into two groups, namely Continue dosing groups and Stop dosing groups, with the continuous treatments or the treatment withdrawal, respectively. Then we examined the estrous cycle, serum T levels, and ovarian morphology.

Excess androgen is the most common symptom of PCOS. About 80%-85% of PCOS women have hyperandrogenemia in clinic [23, 24]. In the DHEA-induced PCOS mouse model fed a normal chow or an HFD, there was a significant increase in serum T levels. Two or four weeks after the withdrawal of DHEA or DHEA + HFD, serum T levels in DHEA mice and DHEA + HFD mice were similar to that in control mice, indicating that the elevated serum T levels were caused by the administration of exogenous DHEA.

Disturbed menstrual cycles are usually associated with abnormal ovulation. In previous research [22, 25], we and others have shown that administration of DHEA fed a normal chow or an HFD leads to an irregular estrous cycle in mice, which corresponds to chronic anovulation or oligomenorrhea in women with PCOS [26]. In this study, the estrous cycle of the mice in Stop dosing groups, including DHEA mice and DHEA + HFD mice, returned to normal 2 weeks after the treatments were stopped. In addition, different from DHEA mice, the estrous cycle of DHEA + HFD mice remained the same as that during the modeling period 1 week after stopping treatments, suggesting that abnormal lipid metabolism may have a certain effect on the estrous cycle. In general, these results implied that it is necessary to continue the treatments with DHEA or DHEA + HFD to maintain the disturbed estrous cycle in mice.

About 40% of PCOS women are affected by infertility [3]. Oligo- and/or anovulation in women with PCOS is likely to be the cause of infertility. In PCOS, infertility may result from the dysfunctional corpus luteum [27]. Consistent with our previous study, DHEA- and DHEA + HFD-induced PCOS mice showed a significant decrease in the number of corpus luteum and an increase in the number of apparent ovarian cysts compared with control mice. Withdrawal of the treatments caused increases in the number of corpus luteum and decreases in the number of ovarian cysts in DHEA mice and DHEA + HFD mice, suggesting the possible recovery of ovulation in these mice.

Women with PCOS are usually accompanied by obesity [28]. Although body weight is not a criterion to determine PCOS, an increase in body weight may reflect disturbed lipid metabolism. DHEA- and DHEA + HFD-induced PCOS mice showed significantly increased body weight compared with control mice. After modeling, DHEA mice and DHEA + HFD mice exhibited normal weight when the treatments were withdrawn. In addition, even with continuous treatments, there was no apparent difference in body weight among control, DHEA, and DHEA + HFD mice 3 weeks after modeling. The reasons remain unknown. One possibility is that DHEA may have differential effects at different ages. For DHEA-induced PCOS mice, prepubertal female mice received DHEA treatments at age 25-day. In girls at puberty, adrenal androgen (including DHEA) secretion increases T secretion and stimulates the development of pubic and axillary hair and acne. For people more than 30 years old, DHEA production has declined. DHEA supplementation decreased fat mass and improved lean body mass and health status in both men and women [29–32]. Another possible explanation is that long-term androgen administration may affect the synthesis of fat or the distribution of adipose tissue in women and lead to weight loss [33]. This phenomenon has been reported in female-to-male transsexuals [34]. These data suggested that DHEA may play a protective role in aging.

In conclusion, after PCOS mice were induced with DHEA or DHEA + HFD treatments, the mice still need consecutive treatments to maintain reproductive phenotypes and meet the diagnostic criteria of PCOS defined by the 2003 Rotterdam criteria. The results were summarized in Table 2. This should be noted when conducting long-term experiments using these mouse models. Indeed, several studies also lengthened DHEA treatment time in long-term intervention experiments [35–37], which ensured the reliability of this PCOS mouse model. Although we did not examine whether similar results would be observed in other postnatal continuing manipulation-induced PCOS animal models, it is recommended to verify whether the animals still exhibit the reproductive phenotypes of PCOS in long-term experiments.

**Table 2:** Summary of reproductive phenotypes of the mice at 2 or 4 weeks after PCOS models were established

	Continue dosing group				Stop dosing group			
	DHEA		DHEA + HFD		DHEA		DHEA + HFD	
	2 weeks	4 weeks	2 weeks	4 weeks	2 weeks	4 weeks	2 weeks	4 weeks
Disturbed estrous cycle	^a^✓	✓	✓	✓	×	×	×	×
Hyperandrogenemia	✓	✓	✓	✓	×	×	×	×
Polycystic ovaries	✓	✓	✓	✓	-	×	-	×
Maintain PCOS reproductive phenotypes	✓	✓	✓	✓	×	×	×	×

^a^✓ consistent, × unconsistent, - unable to judge

### Supplementary Information

**Additional file 1:** **Supplementary Table 1.** General nutrition components of mouse feeds (kcal%).

## Data Availability

The datasets used and/or analyzed in the current study are available from the corresponding author upon reasonable request.

## Abbreviations

PCOS: Polycystic ovary syndrome
DHEA: Dehydroepiandrosterone
HFD: High-fat diet
T: Testosterone
IR: Insulin resistance
TP: Testosterone propionate
DHT: Dihydrotestosterone
AMH: Anti-Müllerian hormone
PFA: Paraformaldehyde
CL: Corpus luteum
H&E: Hematoxylin and eosin
ANOVA: Analysis of variance
