# Supplementary material for: Dehydroepiandrosterone-induced polycystic ovary syndrome mouse model requires continous treatments to maintain reproductive phenotypes
Source: J Ovarian Res. 2023 Oct 25;16:207. doi: 10.1186/s13048-023-01299-8 (PMC10599050; doi:10.1186/s13048-023-01299-8)
Supplement: Supplementary file 1 — Additional file 1: Supplementary Table 1. General nutrition components of mouse feeds (kcal%). [file 13048_2023_1299_MOESM1_ESM.docx]

**Supplementary Table 1**. General nutrition components of mouse feeds (kcal%)

| Nutrition Components | Chow Diet | 60% High-fat Diet |
| --- | --- | --- |
| Protein | 22.47 | 20 |
| Carbohydrate | 65.42 | 60 |
| Fat | 12.11 | 20 |
| Total | 100 | 100 |
